# Small RNA-Seq to Unveil the miRNA Expression Patterns and Identify the Target Genes in *Panax ginseng*

**DOI:** 10.3390/plants12173070

**Published:** 2023-08-27

**Authors:** Chang Liu, Yang Jiang, Ziyi Yun, Kexin Zhang, Mingzhu Zhao, Yi Wang, Meiping Zhang, Zhuo Tian, Kangyu Wang

**Affiliations:** 1 College of Life Science, Jilin Agricultural University, Changchun 130118, China; lchang1205@163.com (C.L.); jiangyangyang1031@163.com (Y.J.); ziyiwing270@163.com (Z.Y.); zkx12302023@163.com (K.Z.); mingzhuzhao@jlau.edu.cn (M.Z.); yi.wang@jlau.edu.cn (Y.W.); meiping.zhang@jlau.edu.cn (M.Z.); 2 Jilin Engineering Research Center Ginseng Genetic Resources Development and Utilization, Changchun 130118, China; 3 College of Information Technology, Jilin Agricultural University, Changchun 130118, China

**Keywords:** *Panax ginseng*, miRNA, target gene, miR166, miR396

## Abstract

*Panax ginseng*, renowned for its medicinal properties, relies on adventitious roots and hairy roots as crucial sources for the production of ginsenosides. Despite the widespread utilization of ginseng, investigations into its miRNAs have remained scarce. To address this gap, two samples of ginseng adventitious roots and ginseng hairy roots were collected, and subsequent construction and sequencing of small RNA libraries of ginseng adventitious roots and hairy roots were performed using the Illumina HiSeq X Ten platform. The analysis of the sequencing data unveiled total miRNAs 2432. The miR166 and miR396 were the most highly expressed miRNA families in ginseng. The miRNA expression analysis results were used to validate the qRT-PCR. Target genes of miRNA were predicted and GO function annotation and KEGG pathway analysis were performed on target genes. It was found that miRNAs are mainly involved in synthetic pathways and biological processes in plants, which include metabolic and bioregulatory processes. The plant miRNAs enriched KEGG pathways are associated with some metabolism, especially amino acid metabolism and carbohydrate metabolism. These results provide valuable insights miRNAs and their roles in metabolic processes in ginseng.

## 1. Introduction

microRNA (miRNA) is a class of endogenous small single-stranded RNA molecules with a length of about 18–24 nt, which mainly plays a regulatory role in the post-transcriptional process of cell differentiation, proliferation, apoptosis, etc., in eukaryotes [1]. A mature miRNA is highly conserved and usually has multiple target genes. Plant miRNA and target gene sequences are almost completely complementary and paired, which can be cleaved after transcription or inhibited by translation to negatively regulate the expression level of target genes [2]. The miRNA plays an important regulatory role in the process of growth and development in plant, metabolism and disease occurrence, and can also be used as biomarkers of diseases or drug targets for clinical disease diagnosis and treatment [3]. Identification and analysis of miRNA have become an important means for scientists to study the function of miRNA bioinformatics. At present, the establishment and development of miRNA quantitative identification methods mainly focus on the comprehensive use of various probe design and labeling technologies, combined with microarray CHIPs, high-throughput sequencing technology, real-time quantitative PCR, and other technologies to improve the detection sensitivity and specificity [4].

The miRNAs in medicinal plants are a class of endogenous small RNAs (sRNAs) that are essential for post-transcriptional regulation of related gene expression. The medicinal plant biosynthesis studies results have shown that the mature miRNAs can be produced either by standard messenger RNA splicing mechanisms or by the process of ribosomal pre-RNA splicing [5]. Computer-assisted high-throughput sequencing technology is the most important method for plant miRNA discovery [6], which is widely used in the discovery and mining of medicinal plant miRNA. Thirty-seven miRNAs were identified and predicted in the transcriptome data of *Vinca minor*, functional analysis of target genes showed that these miRNAs were highly related to the growth and development of *Vinca minor* and the secondary metabolism of terpenoid indole alkaloids, its active components [7]. Two hundred two target genes corresponding to 33 conserved miRNAs and 306 potential target genes corresponding to 92 new miRNAs were predicted in *Eucommia ulmoides*. These target genes are mainly structural and regulatory genes in secondary metabolic pathways, including transcription factors and functional genes in secondary metabolic biosynthetic pathways. [8]. Eleven miRNA families were identified in *Mentha* spp., including 5 first reported miRNA families in Labiaceae, and 130 different target genes of 8 miRNA families were predicted [9]. The leaves of five typical *Hypericum* plants were used for transcriptome sequencing, miRNA and their corresponding target genes were predicted by computer, and miRNA-mRNA interaction analysis was performed. A total of 881 miRNA families and their corresponding target genes were identified [10]. Hundred sixty-eight conserved and 14 non-conserved miRNAs were identified, and the target genes of these miRNAs were predicted in *Astragalus*, which was found that miRNAs of the same family usually have the same expression pattern at low temperatures [11]. Eighty conserved and 41 novel miRNAs were identified. The target gene prediction showed that many miRNAs targeted key enzyme genes and transcription factors are related to artemisinin synthesis in leaves of *Artemisia* [12]. One thousand hundred ninety-seven conserved miRNAs and 2117 novel miRNAs in *Ginkgo biloba* were identified by high-throughput sequencing technology [13]. Fifty novel and 38 conserved miRNAs were identified in *Lycium barbarum* [14]. There were 181 conserved and 173 novel miRNAs, and the target gene prediction results showed that these miRNAs had a total of 519 potential target genes in *Catharanthus roseus* [15].

Ginseng (*Panax ginseng* C. A. Meyer) is a perennial herb in the Araliaceae family. It has been used for many years and has high medicinal value. Ginsenosides, as the main pharmacological active components of ginseng, play a key role in the treatment of many diseases. It has pharmacological effects, such as anti-inflammatory, anti-oxidation, anti-cancer, anti-senile dementia, and anti-atherosclerosis [16]. Ginseng has a long growth cycle, strict requirements for the growth environment, a small stock of wild resources [17], and many problems in artificial cultivation, so it is urgent to use biological engineering methods to develop and protect it. Adventitial roots are products developed from plant organs, such as leaves, roots, and stems, with good genetic and metabolic stability. Induction of adventitious roots in plants has been achieved in several plant species and they can be used to produce high-value secondary metabolites with medicinal, nutritional, and health care functions [18]. *Agrobacterium rhizogenes* induced ginseng explants to produce hairy roots, which have the advantages of fast growth rate, hormone autotrophism, genetic stability, rich secondary metabolites, and high saponins content [19]. It has been used as a bioreactor for the production of secondary metabolites in ginseng [20] and as an analytical material for functional genomics of ginseng [21,22].

In recent years, there has also been miRNA identification in ginseng. Wu et al. [23] identified 73 conserved miRNAs, which can be classified into 33 families from roots, stems, leaves, and flowers of ginseng. Twenty-eight non-conserved miRNAs belonged to nine families and were experimentally validated against eight of them. Among all the predicted target genes, only about 20% were conserved among plant species, while the others seemed to be non-conserved, indicating the diversity of miRNA functions. A total of 71 miRNA families, including 34 conserved miRNAs, 37 non-conserved miRNA families, and 179 target genes of 17 known miRNAs, were obtained from three- and five-year-old ginseng roots. These results indicated that different miRNAs were in ginseng roots of different growth years [24]. Wang et al. [25] identified 3798 miRNAs, including 298 known miRNAs and 3500 potential novel miRNAs from three ginseng samples of Changbai Mountain in China. Expression pattern analysis and target gene prediction of miR166, miR159, and miR396 were highly expressed in the library, which revealed that miRNAs were involved in regulating the metabolic process and growth and development in ginseng.

However, no miRNA studies have been reported in the adventitious and hairy roots of ginseng. In this study, the small RNA libraries of ginseng adventitious roots and ginseng hairy roots were constructed and performed by high-throughput sequencing. A small RNA dataset of 11,448 known and 315,966 novel miRNAs was identified and created. The expression of miRNAs was analyzed, some miRNAs showed specific expression in ginseng. The predicted targets of many ginseng miRNAs are related to metabolism, genetic information processing, and signal transduction. These results provide a basis for the functional analysis of miRNAs and a theoretical reference for the large-scale production of ginsenosides using ginseng adventitious roots and ginseng hair roots.

## 2. Results

### 2.1. Features of miRNA Population in Panax ginseng

To study the miRNAs in the ginseng adventitious roots and ginseng hair roots, two sRNA libraries were constructed using adventitious roots of ginseng and hairy roots obtained by infecting adventitious roots with the *Agrobacterium tumefaciens*. The libraries were sequenced using Illumina sRNA 30 X deep sequencing technology. After combining the sequences from the two libraries, a total of 94,069,090 raw sequence reads. After the removal of contaminants, including low-quality sequences and readings higher than 35 nucleotides and shorter than 17 nucleotides. We used 12,582,185 clean readings of 17 to 35 nucleotides for further analysis. This suggests the existence of a large, diverse, and highly complex population of small RNAs in ginseng.

**Figure 1:**
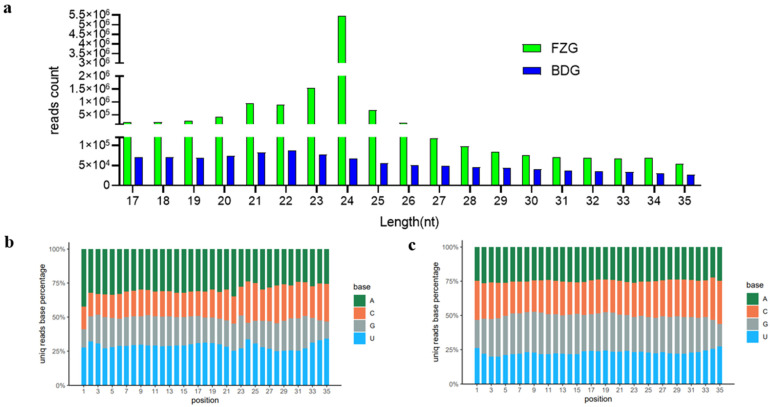
Analysis of miRNA population in ginseng. (**a**) Length distribution and abundance of sRNAs in ginseng adventitious roots (BDG) and ginseng hairy roots (FZG). (**b**) Base distribution in the ginseng adventitious roots. (**c**) Base distribution in the ginseng hairy roots. The horizontal axis is the base position, and the vertical axis is the proportion of a base in the total base at the same position. Different colors represent different bases, and the length of the bar graph represents the proportion size.

Analysis of the size distribution of 12,582,185 miRNA sequences showed that the 24-nucleotide miRNA group was the largest, with approximately (78.69%) of 12,582,185 clean reads being between 21 and 25 nt in length. Among them, 23 (16.46%) and 24 (55.82%) nt were the two main lengths. The clean reading group of 21 bases also accounted for a relatively high proportion (10.32%). The most abundant group of miRNAs was 24 nucleotides (Figure 1a). This suggests that the 24-nucleotide miRNAs are most diverse in ginseng, although their average abundance is low compared to many other studied plants. Among these sRNAs, the percentage of miRNAs starting with A and U nucleotide was highest, and 24-nucleotide miRNAs were enriched for sequences with an A at the 5 ‘end (Figure 1b,c).

### 2.2. Identification of Known and Novel miRNAs

**Figure 2:**
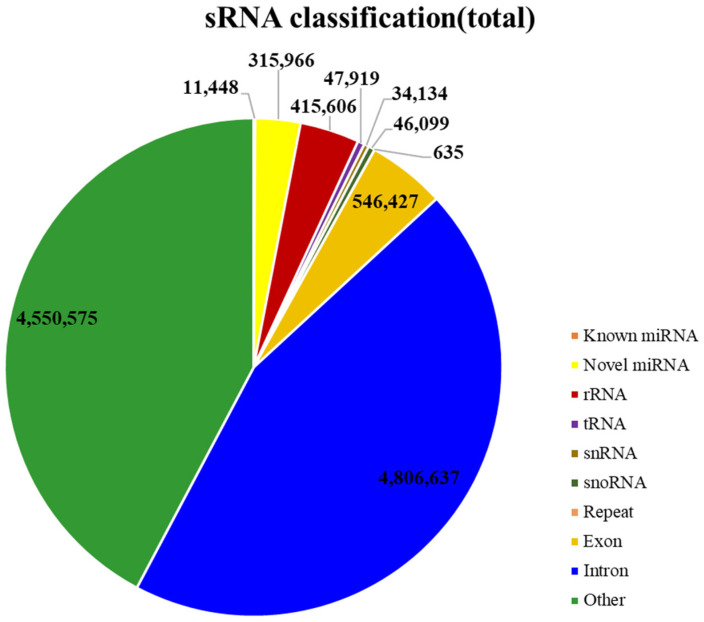
Statistical plot of sRNA classification. The sector in the figure represents the statistical classification of sRNAs, and a larger area of the sector indicates a greater number of sequences of that class. Different colors represent the different kinds.

All clean readings were compared to GenBank, Rfam, and Repbase databases. The sRNA extracted from the two samples of ginseng was divided into different types of RNA, including ribosomal RNA (rRNA), transfer RNA (tRNA), small nucleolar RNA (SnoRNA), and SnRNA. At the same time, the abundance of each type of RNA was determined, and the mature miRNA sequences were mapped to miRbase. We read 11,448 reads of known miRNA mature sequences, and 315,966 novel miRNA reads were obtained after comparison with the ginseng reference genome (Table S1). In addition, the 327,414 reading fragments found in the ginseng adventitious roots and ginseng hair roots were similar to miRNAs previously identified from other plant species. The remainder of the sequences consists of other types of sRNA, including non-coding RNA, tRNA, rRNA, SnRNA, SnoRNA, and novel miRNAs (Figure 2).

### 2.3. miRNAs Differentially Expressed

**Figure 3:**
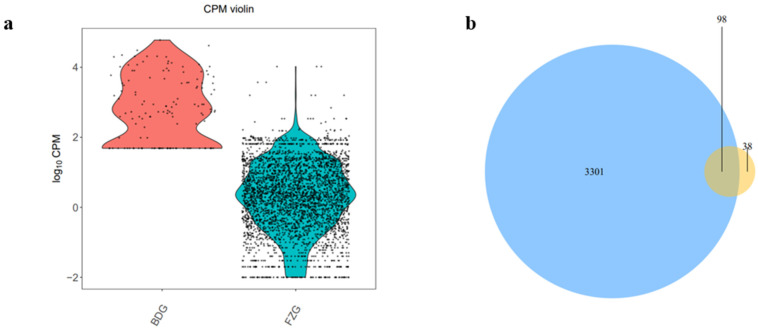
Expression levels of miRNAs in ginseng roots. (**a**) Violin statistics. The x-axis is the sample names, and the y-axis is the log10 transformation value of CPM. (**b**) Co-expression Venn diagram. Different samples are shown in different colors blue for ginseng hairy roots (FZG) and yellow for ginseng adventitious roots (BDG). The numbers represent the number of miRNAs expressed specifically or in common. The overlap region represents the number of expressed miRNAs that are common to different samples, and the non-overlap region represents the number of expressed miRNAs that are unique to different samples.

**Figure 4:**
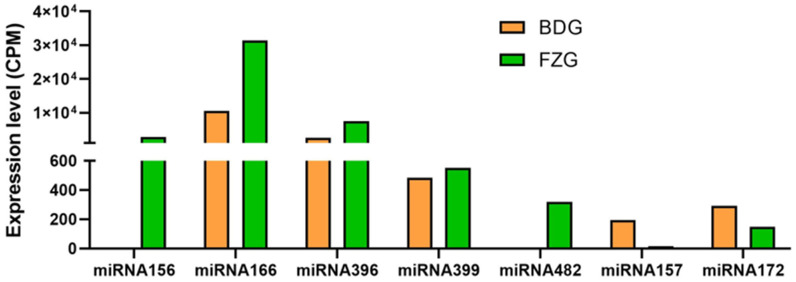
Number of reads for the conserved miRNA family. The units of expression levels were CPM values. Orange is the ginseng adventitious roots (BDG), and green is the ginseng hairy roots (FZG).

Comparative analysis of miRNA abundance between the two ginseng libraries found that the average expression abundance of miRNA in ginseng adventitious roots was lower than that in ginseng hairy roots (Figure 3), but the expression dispersion of miRNA in the two libraries was less, indicating the reliability of miRNA expression determinations. Interestingly, the expression of the miRNA family in hairy roots was 3399, in adventitious roots it was 146, and the miRNA shared by adventitious and hairy roots was 146. After artificial screening of conserved miRNAs, we found that the expressions of miRNA156, miRNA166, miRNA396, and miRNA399 were higher in hairy roots than in adventurous roots. However, the conserved miRNA families were more expressed in the ginseng adventurous roots than the ginseng hairy roots, such as miRNA160, miRNA172, and miR-NA164 (Figure 4).

### 2.4. Target Gene Prediction and Functional Annotation

**Figure 5:**
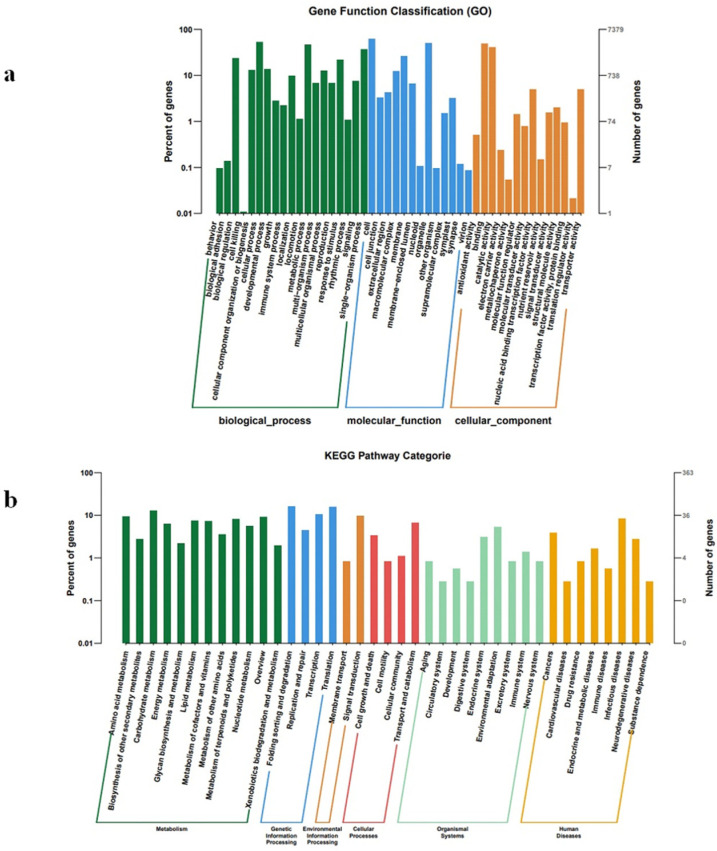
GO function and KEGG annotation of target genes in ginseng adventitious roots and ginseng hairy roots. (**a**) Functional classification of target genes. Green represents biological processes (BP), blue represents molecular functions (MF), and orange represents cellular components (CC). (**b**) KEGG pathway classification of target genes in ginseng adventitious roots and ginseng hairy roots. Different colors represent different classifications.

Prediction of target genes was based on data from identified known and novel miRNAs using Target Finder version 1.0 software. The analysis identified 39,240 targets out of 2432 miRNAs in ginseng (Table S2). To elucidate the biological functions of the identified miRNAs, their possible target genes were analyzed based on Gene Ontology (GO) function annotation. All predicted target genes were divided into 46 functional groups under three GO categories: cell composition (CC), molecular function (MF), and biological process (BP) (Table S2). In the BP group, the most significantly enriched GO terms were cellular process (GO: 0009987), metabolic process (GO: 0008152), and single biological process (GO: 0044699). Within the CC group, the most significantly enriched GO terms were cell (GO: 0005623), membrane (GO: 0016020), and organelle (GO: 0043226). Under the MF group, the most significantly enriched GO terms included catalytic activity (GO: 0003824) and binding (GO: 0005488) (Figure 5a). In addition, to further understand the miRNA target genes functioning in metabolic pathways, the predicted target genes were mapped to the KEGG database and categorized into 271 signaling pathways, of which 11 pathways were significantly enriched (Table S2). It is worth noting that metabolism accounted for the largest proportion in KEGG pathways, among which amino acid metabolism and carbohydrate metabolism had a large number of target genes. The average richness of genes was highest in pathways related to Genetic Information Processing. Two significantly enriched signaling pathways were closely related to Genetic Information Processing and Transcription, including Spliceosome (ko03040) and RNA transport (ko03013) (Figure 5b).

### 2.5. qRT-PCR Verification and Analysis

**Figure 6:**
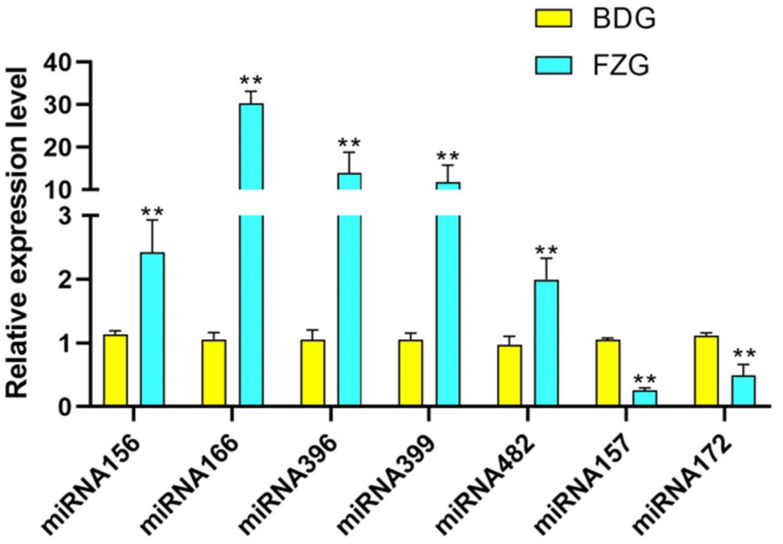
The expression pattern of conserved miRNAs in ginseng was studied using qRT-PCR. The *U6* gene was used as the reference gene. Yellow is the ginseng adventitious roots (BDG), and cyan is the ginseng hairy roots (FZG). Normalized miRNA levels in BDG were arbitrarily set to 1. “**” as significant at *p* < 0.01.

Seven conserved miRNAs were randomly selected for qRT-PCR validation. The results showed that the expression patterns of the selected miRNAs were consistent with those obtained by high-throughput sequencing (Figure 6). The expressions of miRNA156, miRNA396, miRNA166, and miRNA399 were higher in ginseng hairy roots than ginseng adventurous roots, while miRNA172 and miRNA157 were higher in ginseng adventurous roots than ginseng hairy roots.

## 3. Discussion

The miRNAs were a class of non-coding RNAs that played a key role in the regulation of gene expression [26]. High-throughput sequencing and bioinformatics methods are effective means to identify miRNAs [27]. In other plants, many conserved miRNAs have been identified, although only relatively few have been well characterized [28,29,30]. In ginseng, the current research on miRNA is on the roots of different parts of ginseng [23] and different ages [24], and the research on the ginseng adventitious roots and ginseng hairy roots has not been reported. Therefore, we performed high-throughput sequencing on the adventitious and hairy roots of ginseng and obtained the sRNA map of the adventitious and hairy roots of ginseng. This is the first major study of miRNAs in this species. Sequence analysis showed that most sRNAs were between 21 and 25 nucleotides in length. These results are typical of the distribution of sRNA sequences and are consistent with the known 18–25 nucleotide range of miRNAs [31,32], suggesting that the size distribution of SRNA is not uniform. The base at the 5 ‘end is important for small RNA binding to the AGO (Argonaute) proteins complex, and in *Arabidopsis*, most of the 5’ end is uracil U, bound to AGO1. Previous studies have shown that most of the sequences in plants start with U, while 24-nt siRNA-directing DNA methylation and heterochromatin and silencing transposable elements mainly start with A [33,34,35]. Our results indicate that the small first base bias in the adventitious and hairy roots of ginseng is consistent with that of other plants, with the majority beginning with U at 21 nt and the majority beginning with A at 24 nt. The miRDeep2 version 2.0.0.8 software was used to predict the known and unknown miRNAs in the adventurous and hairy roots of ginseng and found a total of 3437 miRNAs.

Adventitious roots grow slowly. On the contrary, hair roots grow rapidly in hormone-free medium [36,37]. Some miRNAs are related to plant growth and development. To explore the differences in miRNA expression between adventurous and hairy roots, in the sequencing results, we randomly selected seven conserved miRNAs and found that miRNA156, miRNA396, miRNA166, the expression of miRNA399 in hairy roots was higher than that in adventurous roots, and miRNA172 and miRNA157 were opposite, indicating that they are closely related to ginseng root growth. In chickpeas [38], osmanthus [39], maize [40], and switchgrass [41], miRNA involved in growth and development have also been studied. GO has a total of three ontologies, which respectively describe the molecular function, cellular component, and biological process of genes [42]. We predicted the target genes of miRNA and performed GO functional annotation of the target genes. All predicted target genes were classified into 46 functional groups under three broad categories: cell composition, molecular function, and biological process. KEGG (Kyoto Encyclopedia of Genes and Genomes) is a comprehensive database of biological systems integrating information on genomes, chemicals, and system functions. Among them, KEGG genes collect all known gene protein sequences of the complete genome and contain minimum information for each gene [43]. A total of 271 KEGG pathways were enriched in the target genes, and 277 genes were closely related to metabolism in these pathways. These results indicate that the functions of miRNA target genes are complex.

We selected several conserved miRNAs as representatives and performed fluorescence quantitative assays to investigate their expression in adventitious and hairy roots. It can be seen that the miRNA expression results of fluorescence quantitative were consistent with the miRNA expression results obtained by sequencing. As reported in previous studies, these miRNAs are involved in plant growth and development, which also proves the accuracy of our experimental results. In this study, we investigated the expression of miRNAs in the adventurous and hairy roots of ginseng to provide some theoretical basis for the future study of ginseng metabolites.

## 4. Materials and Methods

### 4.1. Plant Materials in this Study

Wild-type ginseng adventitious roots (BDG) were obtained by subculture of single-root adventitious roots derived from callus induction in a previous laboratory, and wild-type hairy roots (FZG) were obtained by infection of wild-type adventitious roots with *Agrobacterium rhizogenes* (A4). All plant materials were obtained from the Jilin Engineering Research Center for Development and Utilization of Ginseng Genetic Resources. Immediately after collection of post plant samples, samples were flash-frozen in liquid nitrogen and stored at −80 °C for further analysis.

### 4.2. Library Construction and Sequencing of RNA in Ginseng

RNA was extracted from the adventitious and hairy roots of ginseng, and two libraries were successfully constructed according to standard procedures for small RNA library construction and sequencing [44]. The amplified products were purified by PAGE and sequenced, and the quality of the library was evaluated on an Agilent Bioanalyzer 2100 system [45]. The obtained products were sequenced on the Illumina Hiseq X ten platform [46] with a sequencing depth of 30 × megabits, and the sequencing was completed by Shanghai Sangon Co., Ltd., Shanghai, China.

### 4.3. Quality Control of Sequencing Data

The raw data obtained from sequencing contains lower-quality sequences and splice sequences. To ensure the quality of the information analyzed, the raw data must be filtered and processed using Trimmomatic version 0.36 software [47] and cutadapt. First, the sequence with N base was removed. Splice sequences and low-quality bases were then removed from the reads. Finally, reads less than 17 nt and more than 35 nt in length were removed to obtain clean data. Finally, the repetitive sequences were removed, and unique reads were obtained. These final high-quality data obtained will be used for subsequent analyses.

### 4.4. Identification of Known and Novel miRNAs in Ginseng Adventurous Roots and Ginseng Hairy Roots

For the preliminary analysis, the data were quality controlled, and the length distribution of the small RNAs was finally determined. Then, the Bowtie version 1.1.1 software tool [48] was used to align the clean tags with GenBank, exons, and intron sequences of the reference genome, Rfam, and Repbase databases (mismatch set to less than or equal to 1). Non-coding RNAs (NcRNAs) were excluded, including NcRNAs, such as rRNA, tRNA, SnRNA, snoRNA, and repetitive sequences. The sequences of known miRNAs of the species were obtained from miRBase database [49], and miRDeep2 software [50] was used to identify known miRNAs. The plant mature miRNAs in the miRBase database were searched with the Basic Local Alignment Search Tool (BLAST) to identify the conserved miRNAs in the constructed ginseng small RNA library. Based on the ginseng genome, mirDeep2 version 2.0.0.8 software was used for novel miRNA prediction.

### 4.5. The miRNA Expression Pattern Analysis

The counts of mature miRNAs were quantified, and the counts were normalized to CPM (reads count per million). According to the CPM value of each sample, the Violin diagram [51] was made by R software to show the distribution of CPM of each sample. VENN diagram can be used to count the number of common and unique expressed genes (CPM > 0) in samples. The Venn Diagram package of R version 3.5.3 software [52] was used. The composition similarity and overlap of the number of expressed genes in the samples were visually displayed.

### 4.6. The miRNA Target Gene Prediction

Plant miRNAs were almost completely complementary to their target mRNAs. The miRNAs can bind to any region of the target mRNA (mainly the protein-coding region) by cleaving the target mRNA or inhibiting its translation in the plant. To achieve the regulation of gene expression. For plant miRNAs, Target Finder version 1.0 software [53,54] was used for prediction, and predictions were made using default parameters.

### 4.7. Functional Annotation of miRNA Target Genes

Because multiple transcripts of genes produced by alternative splicing may have different biological functions, we used Blast2GO software version 6.0.3 [55] to annotate and classify predicted target genes in GO. GO annotation and classification results were used to assess functional differences in miRNA target genes. We used the WEGO software (https://wego.genomics.cn/, accessed on 14 April 2023) online tools [56] to visually analyze the results to show the miRNA target gene family gene ontology annotations. Next, the miRNA target genes were mapped to the KEGG database [57] to clarify their involvement in metabolic synthesis and biological processes.

### 4.8. The miRNA Genes Expression Analysis Using the qRT-PCR

Total RNA was extracted from ginseng adventitious roots and ginseng hairy roots using TRIZOL reagent. We hybridized approximately 100 ng of DNA-free total RNA with miRNA-specific stem-loop primers using the stem-loop reverse (Table 1) transcription kit from Bio-7000. The hybridized miRNA molecules were subsequently reversed to cDNA. Amplification was performed with a Bio-7000 real-time polymerase chain reaction system (Bio-Rad, Santa Clara, CA, USA).

Each PCR reaction was performed in a 10 mL final volume containing 10 mL SYBR Green qPCR Master Mix II (Shandong Sparkjade Biotechnology Co., Ltd., Jinan, China). The miRNA-specific primers shown in Table 1 were used. In this study, the cDNA was reverse-admitted from 10 ng total RNA of ginseng adventitious roots and ginseng hairy roots. The following conditions were maintained: 95 °C for 30 s, 40 cycles at 95 °C for 10 s, 60 °C for 25 s, and 72 °C for 25 s. U6 was used as the reference gene for the miRNA in ginseng. The qRT-PCR expression analysis results were analyzed and shown by the 2^−∆∆CT^ [58] method in this study.

## 5. Conclusions

In this study, we collected two samples of adventitious roots and hairy roots of ginseng by small RNA sequencing. A total of 2432 miRNAs were identified for two ginseng samples. The results of miRNA expression pattern analysis were verified by qRT-PCR. Target gene prediction, GO function annotation, and KEGG pathway analysis of miRNA showed that the main functions were related to biological processes, including metabolic process and biological regulation process. Enriched KEGG pathways are associated with some metabolisms, especially amino acid metabolism and carbohydrate metabolism. These results provide valuable insights into the study of ginseng miRNAs and a theoretical basis for studying the involvement of ginseng adventitious roots and ginseng hairy roots miRNAs in secondary metabolism.

## Figures and Tables

**Table 1:** All miRNA-specific primers required of miRNA in ginseng for qRT-PCR.

miRNA and Reference Gene	Stem-Loop Primers	Forward Primer	Reverse Primer
miRNA166	GTCGTATCCAGTGCAGGGTCCGAGGTATTCGCACTGGATACGACGGGGAA	GCGTCGGACCAGGCTTCA	AGTGCAGGGTCCGAGGTATT
miRNA396	GTCGTATCCAGTGCAGGGTCCGAGGTATTCGCACTGGATACGACCAGTTC	CGCGTTCCACAGCTTTCTT	AGTGCAGGGTCCGAGGTATT
miRNA156	GTCGTATCCAGTGCAGGGTCCGAGGTATTCGCACTGGATACGACGTGCTC	CGCGCGTGACAGAAGAGAGT	AGTGCAGGGTCCGAGGTATT
miRNA399	GTCGTATCCAGTGCAGGGTCCGAGGTATTCGCACTGGATACGACAAGGGC	GCGCGCCAAAGGAGAGTT	AGTGCAGGGTCCGAGGTATT
miRNA482	GTCGTATCCAGTGCAGGGTCCGAGGTATTCGCACTGGATACGACGGAATG	GCGTCTTGCCAATTCCTCC	AGTGCAGGGTCCGAGGTATT
miRNA157	GTCGTATCCAGTGCAGGGTCCGAGGTATTCGCACTGGATACGACGTGCTC	CGCGCGTTGACAGAAGATAGA	AGTGCAGGGTCCGAGGTATT
miRNA172	GTCGTATCCAGTGCAGGGTCCGAGGTATTCGCACTGGATACGACATGCAG	GCGCGAGAATCTTGATGATG	AGTGCAGGGTCCGAGGTATT
U6	TTGTCTGACGACGAGAGAGAGCACG	GTGCAGGGTCCGAGGTTTGGACCATTTCTAGAT	TTGTCTGACGACGAGAGAGAGCACG

## Data Availability

The sequences of the miRNA had been submitted to NCBI under BioProject PRJNA1001902, under SRA SRR25505044 and SRR25505045, BioSample SAMN36825377: ginseng adventitious roots, SAMN36825378: ginseng hairy roots.

## Supplementary Materials

The following supporting information can be downloaded at: https://www.mdpi.com/article/10.3390/plants12173070/s1, Table S1: Sequences of all miRNAs identified; Table S2: Target genes of miRNAs as well as GO function and KEGG pathway annotation.
